# Everybody's Page

**Published:** 1892-09-03

**Authors:** 


### Everybody's Page.

The world is better than we thought it. It is stated that five thousand good boys from every nation are to form part of the Chicago exhibit. It is to be hoped their heads won't be turned, for what havoc such a number of bad boys might create in the building!

The establishment in Russia of a separate school of medicine for women which many people had hoped would soon be an accomplished fact has been postponed probably for an indefinite period. It is stated that the project has been set aside through the influence of the Minister of religion, who views the higher education of women in anything but a favourable light. That the study of medicine leads to Materialism and Nihilism is a somewhat remarkable tenet. But every one in Russia lives in such an atmosphere of distrust, that every new project has little chance of success, and Nihilism is suspected where progress only is intended.

The trade in begging has almost reached a science in some countries. In Spain a considerable business, it is alleged, is done in the bartering of children who are infirm, idiotic or deformed. The greater the deformity of the child the better the price which the parent receives for it. In some cases children are made deformed by swathing their legs in bandages so tightly that the circulation is stopped, and atrophy occurs. Superstition has a part in this cruel trade, for children who submit to these malpractices are persuaded to believe that they are gaining Paradise. There is evidently plenty of room for a society for the prevention ot cruelty to children in such a country.

The influence of insufficient ventilation on the spread of consumption is, we venture to think, not yet recognised as fully as it should be. It will be well for the travelling public to bear this in mind. The Marquis of Huntly has recently made some very pertinent remarks upon the state of our railway carriages. There is a Privy Council order providing for the disinfection and cleansing of cattle trucks; is it not time there should be a similar order for the disinfection of the trucks, euphoniously called carriages, which are supplied by some of our lines for passengers? The suburban trains which are constantly going to and fro are seldom if ever ventilated, and, to judge by their appearance, have never known the pleasures of a good wash. It is almost a universal practice as soon as a train has reached its destination to draw up all the windows. The consequence is that by the evening the atmosphere of the carriages on the trains leaving our great cities is absolutely fœtid. Some obnoxious people are in the habit of expectorating freely in railway compartments; this dangerous and offensive habit should be made penal There is no reason why all cushions, those at the back as well as the seats, should not be detachable, so that they can be taken out of the carriages and be thoroughly cleaned periodically and often.

Is the modern quack different from his predecessors? In some respects he probably is, but in his methods rather than in his knowledge. He appeals to a larger circle through the medium of the newspapers than his forefathers, who had to content themselves with the smaller clientele of the poor of his native village, and he has a wider field for the sale of his wares and a greater opportunity for doing harm. Whether he makes more money it is hard to say, certainly his expenses are heavier owing to the need of advertising, but that advertising pays seems clear from the number of quack remedies which are daily dangled before the eyes of the public.

The battle of the mice has been fought over again, but this time not with frogs. A German professor has been their doughty opponent, but he cannot be said to have stood up to them in a very manly way. Professor Loeffler is reported to have discovered a new bacillus (by the way it would be interesting to know how many bacilli have been found during the past few years) which has a deadly effect on mice. To him, accordingly, the Greek Government appealed in order to stay the plague of field-mice which threatened to destroy the harvest in Thessaly. The professor responded to the call, and by means of bread crumbs saturated with his bacillus cultivation, routed the rodents, with whose corpses the fields were soon strewn.

A story comes from America, and is therefore not open to doubt, that a bull recently dashed his horns through the green baize door of a church and carried it up the aisle to the altar, much to the bewilderment of the congregation. Everyone knows the aversion bulls have to red, but it was not usually supposed that any other colour excited the animal's angry passions. Colour-blind people often mistake green and red—can this particular bull be colour-blind? Here is an important subject for American scientists to inquire into, and it is to be hoped a commission will at once sit upon the bull. The scared congregation will, no doubt, readily subscribe to any necessary expenses connected with it.

That cholera is a disease to be feared cannot be denied, but a cholera scare is much more to be feared. Prevention is a potent weapon to fight it with, and everyone should look to his dustbin, be moderate in his habits, and avoid all excess whether of eating and drinking, or of exercise. The excellent example set by Cyrus should also be followed. This cautious monarch when he crossed the Choaspes boiled all the water used for drinking purposes in silver bowls; this precept we fear cannot be followed to the letter, as it is not given to us all to possess such magnificent and luxurious surroundings. But we believe it may be safely asserted that water boiled in the humble kettle of ordinary domestic use will taste as good as that boiled in silver bowls.

